# HTA - An open-source software for assigning head and tail positions to monomer SMILES in polymerization reactions

**DOI:** 10.1186/s13321-025-01098-x

**Published:** 2025-10-28

**Authors:** Brenda de Souza Ferrari, Ronaldo Giro, Mathias B. Steiner

**Affiliations:** 1 IBM Research, Avenida República do Chile, 330, Rio de Janeiro, RJ 20031-170 Brazil; 2 IBM Research, Rd J Fco Aguirre Proenca Km 9 SP101, Hortolândia, SP 13186-900 Brazil; 3 IBM Research, Avenida República do Chile, 330, Rio de Janeiro, RJ 20031-170 Brazil

**Keywords:** Polymers, Cheminformatics, Quantum chemistry, Materials science, Materials discovery

## Abstract

**Abstract:**

Artificial Intelligence (AI) techniques are transforming the computational discovery and design of polymers. The key enablers for polymer informatics are machine-readable molecular string representations of the building blocks of a polymer, i.e., the monomers. In monomer strings, such as SMILES, symbols at the head and tail atoms indicate the locations of bond formation during polymerization. Since the linking of monomers determines a polymer’s properties, the performance of AI prediction models will, ultimately, be limited by the accuracy of the head and tail assignments in the monomer SMILES. Considering the large number of polymer precursors available in chemical data bases, reliable methods for the automated assignment of head and tail atoms are needed. Here, we report a method for assigning head and tail atoms in monomer SMILES by analyzing the reactivity of their functional groups based on the atomic index of nucleophilicity. In a reference data set containing 206 polymer precursors, the HeadTailAssign (HTA) algorithm correctly predicted the polymer class of 204 monomer SMILES, achieving an accuracy of 99%. The head and tail atoms were correctly assigned to 187 monomer SMILES, representing an accuracy of 91%. The HTA code is available for validation and reuse at https://github.com/IBM/HeadTailAssign.

**Scientific contribution:**

The algorithm was successfully applied to data pre-processing by tagging the linkage bonds in monomers for defining the repeat units in polymerization reactions.

## Introduction

Polymers are versatile materials with a wide range of applications [1–9]. Their properties are mainly determined by the way in which the repeat units, or monomers, are connected within the polymer structure. Typically, there are two preferential binding sites per repeat unit, and the respective atomic positions in the structure are labeled “head”and“tail” [10]. During polymerization, repeat units might connect head-to-tail, head-to-head, or tail-to-tail [11]. Depending on how the repeating units are connected, intermolecular interactions between the polymer chains in the material can significantly alter the physical and chemical properties of the polymer [12, 13].

In polymer informatics [14], machine learning (ML) techniques are based on machine-readable representations of a polymer’s repeat units, e.g., the Simplified Molecular-Input Line-Entry System (SMILES) [15, 16]. Polymer-SMILES (p-SMILES) is an extension of SMILES in which symbols such as “*”indicate the polymerization points of the repeat units. Alternative representations include the “Hierarchical Editing Language for Macromolecules”(HELM) [17], the “INternational CHemical Identifier”(InChI) [18], “Boehringer Ingelheim Line Notation” (BILN) [19], and CurlySMILES [20]. BILN is an extension of the HELM notation and can represent complex peptides containing multiple chemical modifications. BILN notation improves some shortcomings of HELM, such as ambiguities when translating to the exact structure or from limited generalizability. In general, string representations are limited to homopolymers and are not suitable for capturing the stochastic nature of polymers, such as encoding randomly branched polymers, as is the case for CurlySMILES. Recently, BigSMILES [21] was applied to represent polymers in string format. In BigSMILES, special characters such as “$”and brackets “$$\langle$$” “$$\rangle$$”indicate the position of head and tail bonds between repeat units. As an advancement, BigSMILES can encode copolymers and enables topological representations of polymeric chains in complex polymers. However, BigSMILES strings provide only a qualitative description of a molecular ensemble [22]. To fully characterize a polymer, a probability and weight must be assigned to each polymer constituent. By providing a standard format for digitalizing data, PolyDAT [22] serves as a quantitative extension of BigSMILES.

Although string-format representations of polymers require the tagging of head and tail atoms within the repeat units, computational tools that automatically identify these positions do not yet exist. The Open Parser for Systematic IUPAC Nomenclature (OPSIN) [23] interprets organochemical nomenclature efficiently by returning as output the SMILES in string format. If an IUPAC polymer name is given as input, OPSIN returns the modified SMILES with head and tail atoms tagged. However, this method is limited to polymers whose nomenclature is already established. The Monomers-to-Polymers tool (M2P) uses known chemical reactions to build polymer chains from monomers [24]. This approach is limited to cases where a comparison of polymer chains and repeat units reveals the positions of heads and tails.

**Fig. 1:**
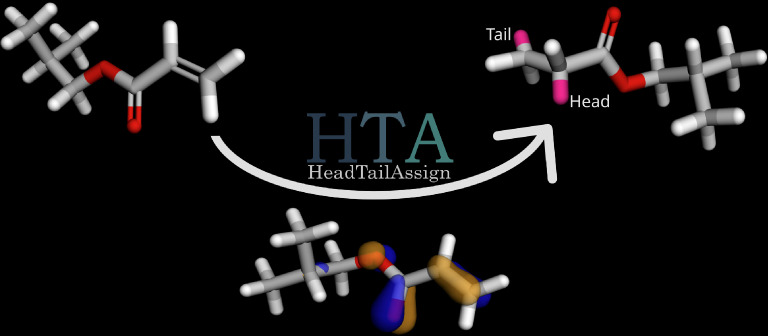
Visual representation of the HeadTailAssign (HTA) method with Poly(isobutyl acrylate) as an example. Isobutyl acrylate is shown on the left and Poly(isobutyl acrylate) on the right. Based on quantum chemical predictions of nucleophilicity, the HTA method identifies the atomic locations at which polymerization reactions occur and assigns head and tail positions. The 3D molecular structure visualization was generated by using RDKit [26]. Starting from a SMILES string, hydrogen atoms were added and conformers were created with a distance-geometry-based conformation generator. Finally, the structure was optimized using the Universal Force Field (UFF) and the canonical molecular orbital HOMO was calculated using PySCF [50–53], using the STO-3 G basis set at SCF / RHF theory level

In this work, we report a method for assigning head and tail atoms in monomer SMILES with the objective to obtain polymer repeat units with bond locations. Our HeadTailAssign (HTA) algorithm quantifies the reactivity of the functional groups of the monomer structure and assigns special characters to those positions in the output SMILES (see Fig. 1). We employed the method in previous work to create a dataset for training ML models that predict polymerization reactions for polyvinyls [25]. In the following, we will introduce the HTA method, discuss the computational workflow, and analyze the performance of the algorithm.

## Methods

### Algorithm

**Fig. 2:**
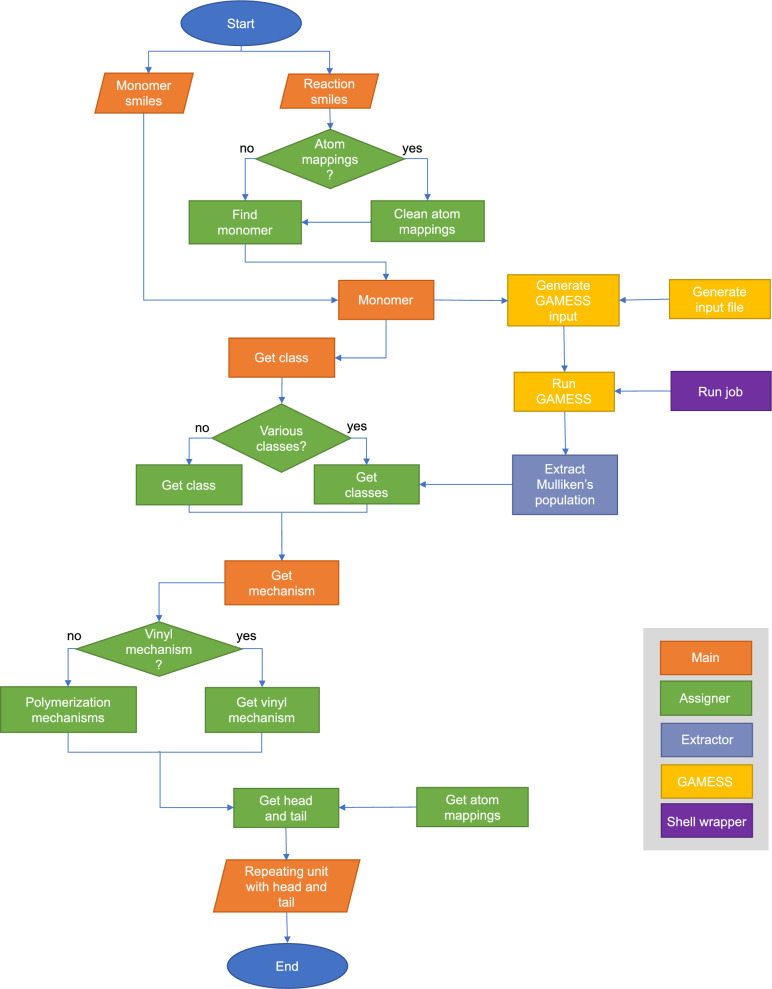
Flowchart of the HeadTailAssign (HTA) algorithm

The HTA algorithm identifies the head and tail positions of the repeat units of a polymer by analyzing the nucleophilicity of its functional groups, see the flow chart in Fig. 2. In short, the algorithm identifies functional groups within the monomer SMILES and rank-orders their reactivity based on quantum chemical calculations. On the basis of this information, the algorithm then assigns the most likely polymerization mechanism, as well as the positions of head and tail atoms.

The input, which is provided as a csv file, contains the polymer name and either a reaction SMILES or a monomer SMILES. If a reaction SMILES is provided, HTA identifies the monomer by evaluating the Chemical Similarity between reactants and products. All molecular entities are separated and their fingerprints are calculated using RDKFingerprint within RDKit [26]. The fingerprints are then compared with Tanimoto Similarity [26, 27]. Finally, HTA selects the reactant SMILES with the highest similarity score as monomer SMILES.

In HTA, data processing is performed by three modules: Assigner, Gamess, and Extractor, see Fig. 2. The Assigner performs classification tasks and tags head and tail atoms. Gamess performs the quantum chemical calculations. The Extractor retrieves information from the output files of the quantum chemical simulations.

The Assigner itself performs three operations: Get Class, Get Mechanism, and Get Head Tail. The operations define the polymer class, the polymerization mechanism, and head and tail positions. For identifying the polymer class, the monomer SMILES is compared with the SMARTS [28] of the most common functional groups that define a polymer class. For example, polyamide contains two functional groups: amide and carboxilic acid. If one of the groups is detected as a nucleophilic site, the molecule is classified as a polyamide.

**Fig. 3:**
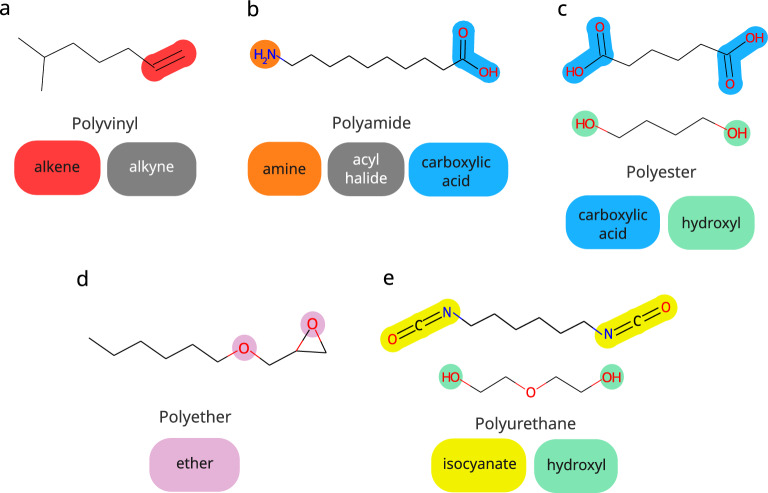
Functional groups for the automated polymer class assignment with the HTA algorithm. Functional groups mapped to (**a**) Polyvinils, (**b**) Polyamides, (**c**) Polyesters, (**d**) Polyethers, and (**e**) Polyurethanes

In the current version (2.1.0) of HTA, Get Class is able to classify five polymerization groups: polyvinyl, polyamide, polyester, polyether, and polyurethane. The functional groups representative of each class are shown in Fig. 3. The most common functional group that promotes polymerization of polyvinyl is alkene. However, in some instances, the alkyne group is also involved.

For polyamide, a copolymer or a cyclic monomer is required for polymerization. We account for this by means of a primary amine and a carboxylic acid, as well as a primary amine and a acyl halide. In the case of a cyclic monomer, we have chosen a secondary amide and a heterocycle monomer to broaden the classification of the polyamide class.

For polyester formation, either a copolymer or a cyclic monomer is required. In the first case, monomers are represented by an aliphatic alcohol group and a carboxylic acid group, respectively. In the second case, they are represented by a heterocycle group and a carboxylic acid group, respectively.

Polyether polymerization requires an opening of a ring in the presence of an ether group. Therefore, the two groups were implemented in HTA to represent the polyether class. Finally, for polyurethane formation, the presence of two monomers is necessary, one with a hydroxyl group, and the other one with an isocyanate group.

If a monomer contains functional groups compatible with multiple class definitions, it is categorized as such. The quantum-chemical calculations then identify the functional group with the highest reactivity, i.e. the most likely polymerization site.

To quantify the reactivity of functional groups within the monomers, we apply the concept of nucleophilicity index, which is based on natural orbitals for atomic populations. The atomic index of nucleophilicity involving the highest occupied molecular orbital (HOMO) is defined as [29]:

1 $$\begin{aligned} R_X=\frac{\sum _\alpha ^X|C_{\alpha ,n}|^{2}}{(1-\epsilon _{n,n})}=\frac{\sum _\alpha ^X|C_{\alpha }|^{2}}{(1-\epsilon ^{\star })} \end{aligned}$$

where $R_X$ is the nucleophilicity index of atom *X*, $C_{\alpha ,n}$ is the Molecular Orbital (MO) expansion coefficient of the $\alpha$th atomic orbital on the *n*th MO, $\epsilon _{n,n}$ and $\epsilon ^{\star }$ are the HOMO energies, *X* is the atom index, $\alpha$ is the index of the atomic orbital, and *n* is the index of the MO.

We calculate the nucleophilicity index $R_X$ with the STO-3G basis set by applying Mulliken’s population analysis method [30–32]. All quantum state functions are calculated at the SCF/RHF theory level using the standard *ab initio* quantum-chemistry package GAMESS US [33], version 2020 R2 and 2024 R2. We selected this combination of basis set and theory level for achieving acceptable accuracy while maintaining computational performance. We also performed the calculation with the 6-31G* basis set and the B3LYP functional to compare accuracy and performance between different theory levels. The test demonstrates that accuracy is maintained using HF/STO-3G (see Results and Discussion section).

We generate the GAMESS input file with the Gamess module. The module converts the SMILES string into a 3D coordinate file using the Python library Pybel [34], a Python wrapper for the OpenBabel [35] toolkit. The 3D coordinate file is generated by OpenBabel with geometry optimization using the classical Universal Force Field [36] (UFF) with 5000 maximum optimization steps. The 3D coordinate file specifies the coordinates and chemical identity of each atom within the monomer. Finally, the Gamess module constructs the GAMESS input file by merging the keywords with the 3D coordinates in xyz format.

The automation of quantum chemical calculations is performed using the run_gamess_job.py script. All variables for running GAMESS are set and then all necessary files are copied to the GAMESS working directory. Next, the command line to run GAMESS is called using the python subprocess module. After the run, the generated output files are transferred to the HTA working directory and all residual files are deleted from the GAMESS working directory.

All information related to Mulliken‘s population of the HOMO is extracted from the GAMESS output file using the Extractor. The module calculates $R_X$ for each of the *X* atoms of the monomer unit and ranks $R_X$ in descending order. In a next step, monomers containing two or more functional groups compatible with existing polymer definitions are classified as follows: the functional group with the highest $R_X$ is identified as the monomer’s polymerization site and the monomer is assigned to the respective polymer class.

The polymerization mechanism is then obtained by using the Get Class routine. The Get Mechanism routine recognizes the class name and assigns a pre-defined mechanism. For instance, if the polymer class is identified as “polyamide”, the routine assigns the “poly-condensation”mechanism to the polymer. This process is straightforward for all classes, except for the vinyl mechanism, in which subcategories exist. For example, a pro-vinyl monomer can polymerize through radical polymerization, cationic polymerization, or anionic polymerization [37]. The likelihood depends on polymerization initiator, solvent, and polymerization stereochemistry.

One way to identify the most likely mechanism subcategory in polyvinyls is to locate the polymerization initiator. If the user provides the reaction SMILES, rather than just the monomer, the algorithm can identify the presence of an initiator related to a specific subcategory. In this case, the algorithm searches among the reactants for the presence of an initiator from a list of initiators, represented by their SMILES strings. To that end, the algorithm compares the chemical similarity of all reactants with all listed initiators using RDKit’s FingerprintSimilarity function. If the similarity score is 1.0, the subcategory to which the initiator belongs is assigned as polyaddition subcategory. The list of initiators is located in the _get_vinyl_mechanism() method in assigner_helper.py file.

After classes and mechanisms are assigned, the HTA algorithm identifies the positions of the head and tail atoms in the monomer SMILES, which are labeled with the symbols “*:1” for head and “*:2” for tail, respectively. For each polymer class, the algorithm contains information about the organic function in which the most nucleophilic atom is located. For example, in case of vinyl polymers, polymerization should occur at the double bonds and, in some cases, at the triple bond. Using the atom mappings, the nucleophilic atom is selected as head by convention. In the case where the electrophilic atom occurs in the same organic function, which is the case in vinyl polymerization, the tail is selected from the same organic function. In polyamides, the tail atom is located within a different organic function. In that case, HTA selects the organic functions with the electrophilic atom and, by using atom mappings, assigns the tail atom accordingly. For some classes, such as polyethers and polyamides, the monomers may be structured as a cycle or a macrocycle. In those cases, the cycle is opened by SMILES manipulation, and structural errors are checked with a dedicated sanitization process. SMILES sanitization ensures that a valid molecular structure can be generated from a SMILES string [38]. In this work, we use the term “sanitization”in the context of manipulating SMILES strings using Regex patterning [39].

The SMILES representation is treated as a sequence of letters, without considering the connections between the atoms. For molecules with aliphatic structures, such as vinyl precursors, this simplification leads to acceptable results. However, for complex molecules, such as cyclic precursors, the connections between atoms should be accounted for.

Finally, the HTA results are compiled in csv format. The files contain polymer name, reaction with and without atom mappings, assignment results for monomer, polymer class, polymerization mechanism, as well as head and tail atoms.

### Data

The validation data set contains 206 data entries in total, with 149 polymers in the vinyl class, 17 in the polyamide class, 25 in the polyester class, 12 in the polyether class, and 3 in the polyurethane class.

We found 57 polymer names with polymer SMILES that belong to the polyamide, polyester, polyether, polyurethane class, respectively, at Polymerdatabase.com [40, 41]. 149 polymer names and (some of the) polymer SMILES that belong to the vinyl class were taken from reference [42]. To complete the data entries, we created the missing polymer SMILES either from scratch or, alternatively, by conferring OPSIN [43]. For validation of the HTA algorithm, we modified the data set by transforming polymer products into precursors, or monomers, on a case-by-case basis. The algorithm could then be tested for detecting the reaction centers of polymerization.

### Validation

For HTA validation, we tested monomers belonging to the following classes: polyamide, polyester, polyether, and polyurethane. In addition, we considered monomers that undergo vinyl polymerization.

Specifically, we compared the true head and tail positions of SMILES with the positions predicted by HTA. The head and tail positions were considered as unique tokens and the difference between heads and tails was not taken into account.

To compare the results for homopolymers, both the ground-truth and the predicted data set were sorted by polymer name, and monomers with heads and tails assigned (mon-HTA). The canonicalization of the SMILES was performed using RDKit, assuring that the labeling is unambiguous. The comparison of the SMILES strings revealed if each mon-HTA entry had the same canonical SMILES in the ground-truth and the predicted data set. Since the number of entries in the validation data set was small, we visually compared each individual molecular structure. The results were compiled as a Boolean series in the HTA output file, while the ground-truth and predicted structures were visualized as a png image file.

## Results and discussion

We validated the performance of the HTA algorithm; see Fig. 2, with a data set containing 206 polymer precursors. The validation data set is described in the Methods section, and the link to the data repository is provided in the Data Availability section.

We first discuss the computational efficiency of the HTA algorithm. Performing the HTA assessment of the full data set required a compute time of roughly 40 minutes on a personal computer (11th Gen Intel Core I5-1135G7, Intel Iris Xe Graphics, 16Gb memory DDR4). This corresponds to about 10 seconds per monomer SMILES, including the quantum-chemical simulations.

For studies of larger datasets, 10 seconds per polymer could become a performance bottleneck. In such cases, we suggest running HTA using HPC infrastructure and/or performing tests at a lower level of quantum theory. To improve the scalability of HTA, we suggest parallelizing the quantum calculations and running GAMESS on GPU. This should be feasible as the quantum calculation for each polymer is an independent process and because GAMESS can be accelerated by GPUs, which could potentially decrease the run time by 10x with Pascal GPUs and 8x with Volta GPUs [44]. By exploring the independent characteristics of each polymer calculation, it would be possible to split the input dataset into subsets to run on different CPUs, and merge the outputs into a single file. In our HPC cluster, it took around 5 seconds to process one polymer at a HF/STO-3G quantum theory level, using one CPU. In an HPC environment equipped with 64 CPUs, it would be possible to compute about 1,000,000 polymers in less than one day.

**Fig. 4:**
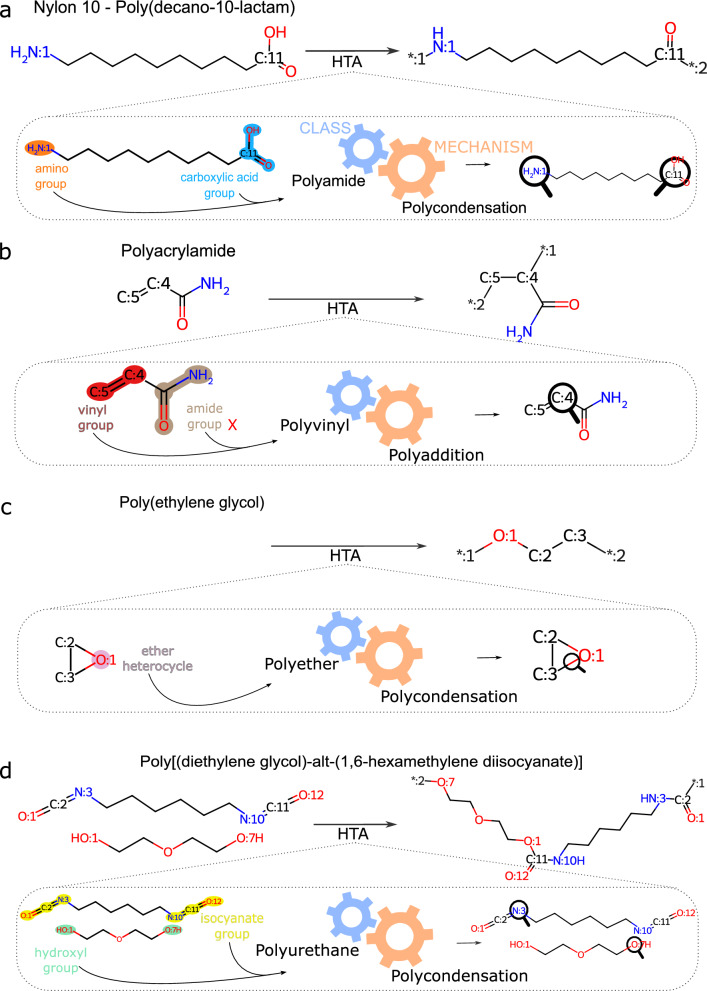
Representative examples of automated polymer classification and head/tail assignments with the HTA algorithm.** a** Nylon10 - Poly(decano-10-lactam), (**b**) Polyacrylamide, (**c**) Poly(ethylene glycol), and (**d**) Poly[(diethylene glycol)-alt-(1,6-hexamethylene disocyanate)]. The symbol “*:1” indicates head atoms, the symbol “*:2” indicates tail atoms. Different colors indicate different functional groups: orange - amino, blue - carboxilic acid, red - vinyl, brown - amide, pink - ether heterocycle, yellow - isocyanate, green - hydroxyl

In Fig. 4, we present four polymer classification examples representing Polyamide, Polyvinyl, Polyether, and Polyurethane. With a reaction SMILES as input, the algorithm performs an initial assessment of Chemical Similarity. Because the validation data set does not contain any polymerization reactions, the algorithm continues with the polymer classification task.

In the first example, the monomer polymerizes to Nylon 10 by means of a polycondensation process (Fig. 4a). The algorithm’s Assigner routine identifies two functional groups in the monomer: an amino group and a carboxylic acid group. By accessing the dictionary that maps the functional groups to polymer classes, the algorithm verifies that both groups indicate the polyamide class and the polycondensation mechanism. The head assignment is performed by finding the atom mapping for the nitrogen of the amino group and the tail assignment is performed by finding the atom mapping for the carbon of the carboxylic acid group.

In the second example, the HTA algorithm detects a vinyl group and an amide group (Fig. 4b). On the basis of the functional group selections that define each class, the HTA algorithm cannot match the monomer with a single polymer class. Therefore, the algorithm has to prioritize the functional groups for polymer class assignment. In this case, the vinyl group is selected. The first reason is that the vinyl group has a higher nucleophilicity index. The second reason is that the amide group is not mapped alone to any polymer class implemented in HTA. Finally, the head and tail positions are assigned to the carbon atoms forming the double bond in the vinyl group, and the structure is sanitized accordingly.

In the third example, the monomer is an epoxy heterocycle (Fig. 4c). For assignment of head and tail, the monomer has to undergo a ring-opening process. Because there is only one functional group in the SMILES string, the assignments of both polymer class and mechanism are straightforward. The head and tail atoms are then assigned as in the previous example.

In the fourth example, a copolymer is represented with polyurethane precursors (Fig. 4d). In this case, the algorithm identifies the relevant functional groups of each monomer, i.e. the isocyanate groups and hydroxyl groups, and groups them together for assigning the polymer class. The algorithm can now identify that those monomers belong to the polyurethane class and polymerize through polycondensation. The heads and tails are then assigned to each monomer as polymerized entities.

**Fig. 5:**
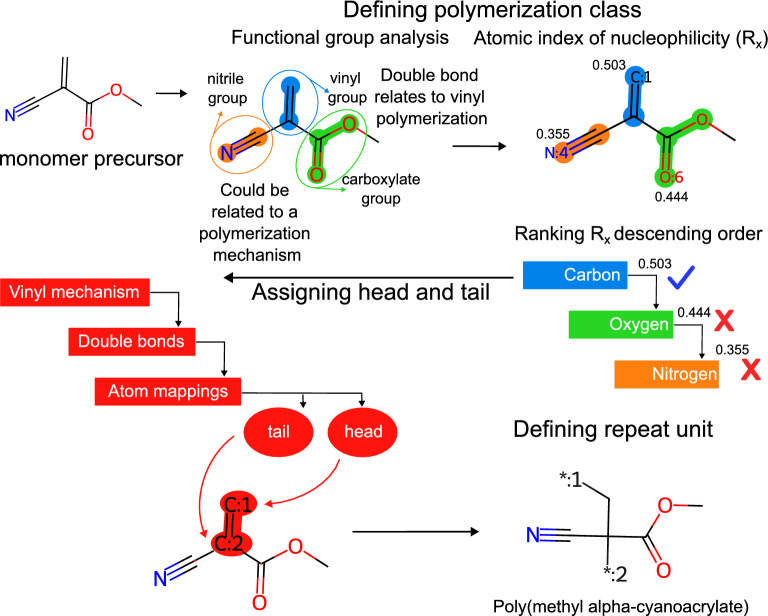
Schematic example of HTA assignment of a polyvinyl. The monomer precursor is analyzed with regards to its organic functions. If the algorithm finds more than one functional group, it will use the atomic index of nucleophilicity, $R_X$, for identifying the site with highest nucleophilicity. Both head and tail are then assigned accordingly

In Fig. 5, we show schematically the HTA-processing of a polyvinyl. The monomer precursor is analyzed on the basis of its organic functions. The analysis is performed using SMARTS patterns located in the assigner.py file. If the analysis identifies more than one functional group that could be related to different polymerization mechanisms, the algorithm uses the nucleophilicity index, $R_X$, to determine the most probable polymerization site. The algorithm identifies the atom with the highest nucleophilicity, the polymerization mechanism, and assigns head and tail using atom mappings. Then, the algorithm performs a sanitization process and produces the SMILES structure.

**Fig. 6:**
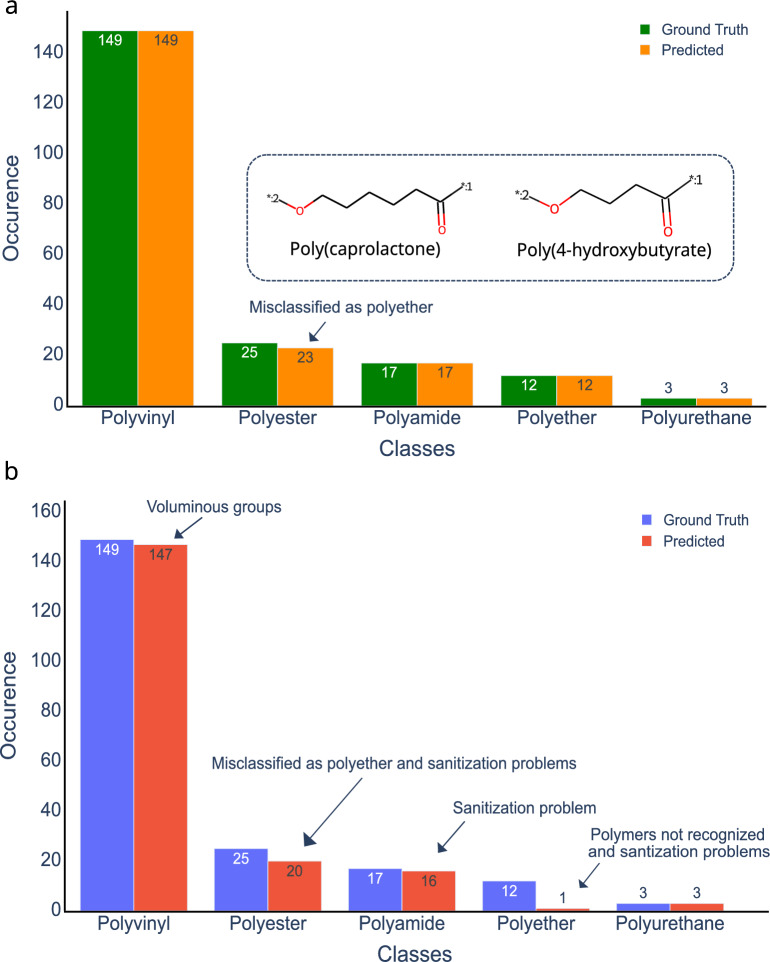
Comparison between HTA predictions and ground truth data.** a** Predicted polymer classes (orange) and ground-truth data (green). An example of an incorrect HTA prediction (miss-classification) is shown with the respective canonical SMILES representation.** b** HTA-based head and tail assignments (red) and ground-truth data (blue). The symbol “*:1” indicates head atoms, the symbol “*:2” indicates tail atoms

We validated the HTA predictions by comparing them with the ground truth and the results are shown in Fig. 6. Of the 206 polymer precursors in the data set, HTA correctly predicted the polymer class for 204, which represents an accuracy of 99.0% (Fig. 6a).

The two monomers of the polyester class that were misclassified, poly(caprolactone) and poly(4-hydroxybutyrate), are displayed in the inset of Fig. 6a. Both precursors are heterocycles; however, the algorithm identified them as cyclic monomers and assigned them to the polyether class. Consequently, the head and tail positions were incorrectly assigned as well. To improve prediction accuracy, a future version of the HTA algorithm should include a definition that cyclic precursors can generate polyester oligomers.

The validation of the head and tail assignment shown in Fig. 6b reveals that the algorithm correctly assigned the positions in 187 cases, representing an accuracy of 90.8%. Within the polyurethane class all monomers were correctly assigned.

**Fig. 7:**
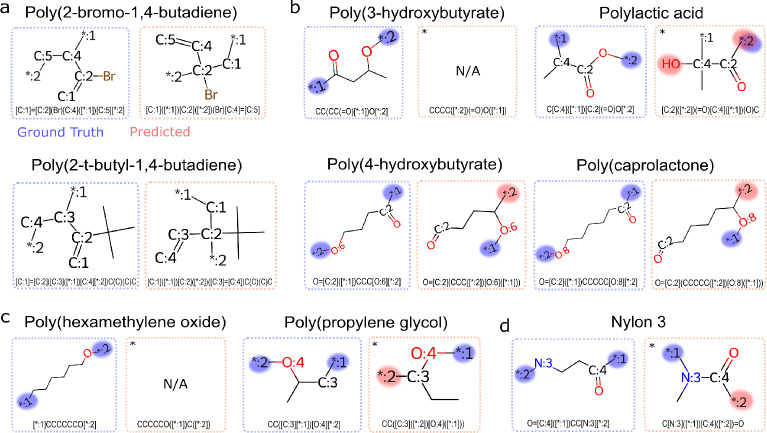
Representative examples of incorrect head/tail assignments by the HTA algorithm in the class of (**a**) Polyvinyl, (**b**) Polyester, (**c**) Polyether, and (**d**) Polyamide. The symbol“*”in the canonical SMILES representations indicate incorrect structure sanitization. The symbol “*:1” indicates head atoms, the symbol “*:2” indicates tail atoms. N/A indicates not applicable, since no valid molecular structure was generated

Within the polyvinyl class, incorrect head and tail assignments occurred in two of 149 monomers. A possible explanation is the presence of large groups connected by one of the double bonds in their structures. Polymerization of vinyl monomers follows the polyaddition mechanism in which the reactive site, i.e. the double bond, is attacked by an initiator. The initiator breaks the double bond by forming a single bond with one of the carbon atoms. The second carbon atom remains available to grow the polymer chain [45]. The attack of the double bond follows chemical rules, and there are situations in which the most nucleophilic atom is not available as a reactive site. As shown in Fig. 7a, the structures of Poly(2-t-butyl-1,4-butadiene) and Poly(2-bromo-1,4-butadiene) contain t-butyl and bromine groups, respectively. These groups act as electron donors, increasing the electron population of the vicinal carbon atoms. However, because of their voluminous nature, they might also increase the steric hindrance in the region. The current version of the HTA algorithm does not incorporate specific rules for predicting steric hindrance. As a result, the head and tail positions were simply assigned to the region with the highest nucleophilicity.

To increase the prediction accuracy for polymers with voluminous groups, we suggest implementing in HTA a site-based, steric hindrance index for reactivity predictions. To evaluate if the position selected by means of highest nucleophilicity is available for a nucleophilic attack, one could use the percent buried volume descriptor [46]. This descriptor quantifies the percentage of the total volume of a sphere occupied by a ligand. It is conceivable that one could calculate the percent buried volume of the ligands linked to the most nucleophilic atom, and check if any of the ligands could hinder a nucleophilic attack. If a nucleophilic attack is hindered, then the second most nucleophilic site could be analyzed as a potential head position and another round of evaluation of the percentage of the total volume should be performed for the ligands in this position. This process would continue until the algorithm identifies a suitable nucleophilic site. The percent buried volume descriptor is implemented in SambVca tool [47] and could be implemented in HTA using MORFEUS [48].

In some cases, we observed that the head-and-tail assignment is correct but the sanitization of the SMILES structure is incorrect, in particular if a ring-opening process is involved. We show the example of poly(3-hydroxybutyrate) polymer with its heterocycle precursor in Fig. 7b). The head and tail positions were correctly assigned to the oxygen atom of the oxetane ring and to the carbon atom of the carbonyl group. However, the ring-opening process performed by the HTA algorithm generated an incorrect SMILES that cannot be visualized. The generated SMILES (“CCCC(=O)([*:2])O([*:1])”) contains one carbon atom with valence 5, while the actual SMILES (“CC(CC(=O)[*:1])O[*:2]”) separates the carbonyl using parentheses to ensure that the carbon atom presents valence 4. We note that it was not possible to resolve this issue using SMILES string manipulation.

In another example, the polylactic acid polymer was correctly classified as polyester (Fig. 7b). The polymer head was correctly assigned to the carbon atom next to the hydroxyl group. Although this carbon atom was correctly identified as the most nucleophilic site, the sanitization process did not remove the hydroxyl group from the tail position, leading to an incorrect sanitization. In addition, the sanitization process removed the oxygen atom from the carboxyl group, transforming it into a carbonyl group. Although this issue occurred in almost all of the polyester structures, we did not consider this to be an incorrect assignment. After all, the positions of head and tail atoms are correct, besides a missing oxygen atom. However, the issue should be addressed in a future update of the sanitization routine.

Such sanitization issues occurred mainly in the polyether class, in which all precursors, except polyacetal, are cyclic structures. As can be seen in Fig. 6b, over 90% of the structures were incorrectly assigned due to issues associated with the ring-opening process. In case of the poly(hexamethylene oxide) polymer (Fig. 7c)), the sanitization process did not produce a SMILES structure suitable for visual inspection. As for poly(3-hydroxybutyrate), the generated SMILES (“CCCCCO([*:1])C([*:2])”) contains one carbon atom with valence 5, while the actual SMILES (“[*:1]CCCCCCO[*:2]”) presents a carbon atom with valence 4. As before, it was not possible to resolve this issue using SMILES string manipulation.

We have observed improper SMILES sequences in two cases and improper ring-opening processes in eight cases. In the case of poly(propylene glycol) shown in Fig. 7c, the sanitization step has generated a proper SMILES structure; however, the ring-opening process was performed in a manner that led to an incorrect assignment of the tail position. When comparing the generated SMILES (“CC(C([*:2])O([*:1]))”) with the ground-truth (“CC(C[*:1])O[*:2]”), the tag located at the oxygen atom is correct but the sanitization procedure opened the ring at the side of the methyl substituent, which is the region most populated by electrons. In fact, bond breaking should occur at the opposite side. The result is an incorrect assignment of the tail position.

The same issue was observed in the sole instance in which an incorrect assignment occurred within the polyamide class (Fig. 7d). In this example, the ring in the precursor of Nylon 3 was incorrectly sanitized and generated a false structure with the amide bond intact. By analyzing the predicted SMILES (“CN([*:1])C([*:2])=O”), we realize that one carbon atom was deleted from the ground truth sequence (“O=C([*:1])CCN[*:2]”).

In general, the opening of the heterocyclic ring is the most significant challenge of the validation process.

Future extensions of the HTA algorithm will require a robust sanitization process for complex monomers, such as cyclic precursors. A potential pathway could be the representation of precursor molecules as graphs during the sanitization phase, with atoms designated as nodes and bonds as edges. The graph representation would allow for the assignment of the bond to be broken, indicated by the edges to be deleted. In addition, atoms or groups of atoms could be deleted or added by indicating the respective nodes. In a straightforward approach using RDKit, a process could be implemented for detecting the location of the rings and the indices of the bonds that belong to the ring. Then, the position of the head could be assigned by checking the atom in which the HOMO is located based on the nucleophilicity analysis. From there, the bond that should be broken or the atom/group that needs to be deleted could be detected by the specific index already defined with RDKit. A graph-based approach could be developed using more general software such as Networkx [49]. In this way, the implementation could be tailored to the specifics of the ring opening process related to the polymerization mechanism. For enhancing the accuracy of the head and tail assignment, we suggest considering the HOMO and the lowest unoccupied molecular orbital (LUMO) as the nucleophilic and electrophilic sites, respectively. In addition, considering the comprehensive chemical information provided by the frontier orbitals may be beneficial.

It is important to note that the quantum theory level used in the validation process might be too low for some applications. In the present case, we also performed a second run with the same dataset using B3LYP/6-31 G* level for evaluating accuracy and performance using HPC infrastructure. By analyzing the results, we observed that the accuracy was maintained at 91% while the performance decreased 157 fold, from 0.16 cpu hours using HF/STO-3 G level to 25.2 cpu hours using B3LYP/6-31 G* level. Therefore, we recommend testing different theory levels with HTA depending on accuracy needs.

Despite the methodological limitations discussed above, the lack of polymer data outside the polyvinyl class has posed a severe limitation for test and validation of the HTA algorithm. By making the initial data set publicly available, we hope that the computational chemistry community will contribute more data to each polymer class. Also, we encourage the community to share information about the precursors in publicly available datasets. In addition, improving the existing chemical rules and adding new polymerization classes should further enhance the usefulness of the HTA algorithm.

## Conclusion

We reported HTA, an algorithm for assigning head and tail atoms in monomer SMILES based on the reactivity of their functional groups. The algorithm can, for example, be applied to data pre-processing in ML by tagging the linkage bonds in monomers for defining the repeat units in polymerization reactions. [25]

In a reference data set of monomer SMILES, the HTA algorithm correctly predicted the polymer class with an accuracy of 99%. The head and tail atoms were correctly assigned with an accuracy of 91%. The HTA performance did not change for different runs and we did not observe significant changes in head and tail assignments as well as polymer class predictions. The geometric optimization of monomers was found to be robust, maintaining the site-specific assignment of nucleophilicity across different runs.

In the validation study, the class prediction for polyester underperformed, as two structures were misclassified as polyether. With regards to the prediction of head and tail positions, all polymer classes presented reasonable accuracy, with the exception of the polyester class. In that case, the low accuracy was due to issues related to the ring-opening process of the polymerization. In the other classes, errors in head and tail assignments were related to limitations with treating steric hindrance and computational sanitization problems.

Future extensions of the HTA algorithm could overcome those limitations. The implementation of a robust SMILES sanitization process for complex monomers, including a graph-based ring opening process, is required.

For enhancing the accuracy of the head-and-tail assignments, we suggest including an analysis of LUMO and frontier orbitals in the quantum chemical simulation process. A refinement of the implemented chemical rules and the addition of new polymerization classes should lead to further HTA performance enhancements. To overcome the data bottleneck, we encourage researchers to contribute more data to each polymer class of the initial data set.

## Data Availability

The data set “input.csv” for validating the HTA algorithm is available under the 10.24435/materialscloud:2x-9j at: https://archive.materialscloud.org/record/2025.120. The HTA output files using different levels of quantum theory and basis sets to calculate the nucleophilicity index with HF/STO-3G and B3LYP/6-31G* “output_hta_hf-sto3g.csv” and "output_hta_b3lyp-631g.csv" - respectively, are available under the doi:10.24435/materialscloud:2x-9j at: https://archive.materialscloud.org/record/2025.120.

## Abbreviations

ML: Machine learning
SMILES: Simplified molecular-input line-entry system
p-SMILES: Polymer-SMILES
HELM: Hierarchical editing language for macromolecules
InChI: INternational CHemical Identifier
BILN: Boehringer Ingelheim line notation
OPSIN: Open parser for systematic IUPAC nomenclature
IUPAC: International union of pure and applied chemistry
M2P: Monomers-to-polymers tool
HTA: HeadTailAssign algorithm
HOMO: Highest occupied molecular orbital
LUMO: Lowest unnocupied molecular orbital
MO: Molecular orbital

## Code availability

The HTA source code is available at https://github.com/IBM/HeadTailAssign.
